# Gone with the flow: Whitefish egg drift in relation to substrate coarseness under a range of flow velocities

**DOI:** 10.1111/jfb.15923

**Published:** 2024-09-02

**Authors:** Topi K. Lehtonen, Lari Veneranta

**Affiliations:** ^1^ Natural Resources Institute Finland Oulu Finland; ^2^ Natural Resources Institute Finland Vaasa Finland

**Keywords:** constructed rivers, habitat, hydro peaking, hydroelectricity, riverbed, salmonid

## Abstract

Most major rivers in Europe have been dammed for hydropower or other purposes. Such river alterations have decimated natural reproduction of many migratory fish species, including that of the anadromous European whitefish, *Coregonus lavaretus*, which is now maintained by extensive stocking programmes. In addition to stocking, limited natural reproduction may occur downstream of dams, where peak flow bouts from the dams threaten to flush the eggs into unsuitable habitats. Here, we assessed the effects of water flow velocity and substrate coarseness on downstream drift of whitefish eggs under laboratory conditions. The experiment's two different gravel substrates retained eggs better than cobble or sand substrates; the water velocity needed for notable egg drift was higher for the gravel substrates. The results indicate that egg drift is one of the factors that should be considered when evaluating the effects of hydropower plant operations. Moreover, measures mitigating the effects of the artificial flow regimes should incorporate the type and coarseness of the riverbed's substrate.

## 1 INTRODUCTION

Globally, hydropower plants with dams are major electricity producers that are considered to be relatively climate friendly (Bao et al., 2023; Berga, 2016). Besides hydropower, river dams are used for flood control, irrigation, and other types of water supply (Wang et al., 2021). Consequently, this anthropogenic environmental impact is ubiquitous, with only a small percentage of major rivers not being affected (Dynesius & Nilsson, 1994; Grill et al., 2015; Nilsson et al., 2005; Schinegger et al., 2012). Yet, hydropower construction is projected to further increase in the future (Grill et al., 2015; Hermoso, 2017; Poff & Schmidt, 2016; Zarfl et al., 2015, 2019). Growing needs for electricity aside, river construction has multiple negative ecological consequences, arising from alterations to flow regimes, increased flux of small particles, altered temperature profiles, decreased water quality, habitat type alterations, and discontinued access of river fauna to their key habitats (Best, 2019; Maavara et al., 2020; Poff & Schmidt, 2016; Schinegger et al., 2012; Schmutz et al., 2015; Zarfl et al., 2019). These changes have had negative effects on many fish populations and communities (Cooper et al., 2017; Liermann et al., 2012; Pringle et al., 2000; Schmutz et al., 2015; Young et al., 2011).

Of the dam operation impacts, changes to natural water flow patterns are often particularly challenging (Nislow & Armstrong, 2012; Schmutz et al., 2015; Warren et al., 2015; Young et al., 2011), especially to the sensitive early life‐history stages of fish, including eggs (Kemp et al., 2011; Liu et al., 2021; Warren et al., 2015). Constructed rivers tend to have pronounced spatial and temporal flow variation, with the amplified flow peaks considerably affecting river ecology (Bruder et al., 2016; Buddendorf et al., 2017; Murchie et al., 2008; Vehanen et al., 2020). Although fish eggs may benefit from high dissolved oxygen levels associated with swift water currents (Bardonnet, 2001), artificially strong flow can also flush the eggs into unfavorable areas, where they may face adverse biotic (e.g., predation) or abiotic (e.g., burial and low oxygen levels) conditions (George et al., 2015; Warren et al., 2015). Notably, such impacts of the peak flow events are likely to depend on the characteristics of the river and riverbed (Lane et al., 2018; Schmutz et al., 2015). For instance, the substrate type might affect the likelihood of the eggs washing out or their subsequent suffocation by the sediment (Acornley & Sear, 1999; Bardonnet, 2001; Payne & Lapointe, 1997; Silva et al., 2015). Therefore, the (peak) flow velocities and river characteristics can have interactive impacts on fish eggs (Buddendorf et al., 2017; Schmutz et al., 2015; Worthington et al., 2014).

The effects of peak flow events on fish eggs are likely to be species‐specific (Chapman et al., 2014; Guo et al., 2023; Tomczak et al., 2024), depending on, among other things, the eggs' physical characteristics and the way they are deposited (Guo et al., 2023; Murray et al., 2013; Riehl & Patzner, 1998). In this regard, flow velocity effects have been studied predominantly in species with semi‐buoyant eggs, for which downstream drift is a natural component of the life history (e.g., Dudley & Platania, 2007; Liu et al., 2021; Zeng et al., 2019). In contrast, much less is known about the effects of water flow on heavier or adhesive eggs, which are not extensively drifting downstream under typical natural conditions. However, such eggs can also be markedly affected by the flow, as in the European river lamprey (*Lampetra fluviatilis* L. 1758) (Silva et al., 2015) and *Acipenser* sturgeons (Yi et al., 2022). Further knowledge about flow effects on eggs is acutely needed for these and other river‐spawning species, such as anadromous populations of the European whitefish (*Coregonus lavaretus* L. 1758).

*Coregonus* species (i.e., whitefishes) differ from most other salmonid fishes in that they do not construct spawning redds, which would provide some protection to the eggs from external conditions (Reshetnikov & Bogdanov, 2011). Instead, spawning takes place in the water column in late autumn so that the eggs are scattered across the spawning site (Fabricius & Lindroth, 1954; Semenchenko & Smeshlivaya, 2021; Ventling‐Schwank & Livingstone, 1994). Eggs flushing away from the spawning site can be a major driver of egg mortality in whitefishes (Etheridge et al., 2011; Murray et al., 2013; Reshetnikov & Bogdanov, 2011; Ventling‐Schwank & Livingstone, 1994). The extra mortality can be critical, given that, similar to many other fish species, egg survival is a significant bottleneck to recruitment in whitefishes (Brown et al., 1991; Brown & Scott, 1994). The issue is particularly relevant in the anadromous, river‐spawning ecotype of *C*. *lavaretus* in the northern Baltic Sea area, where their natal rivers are blocked by dams, with only very limited natural reproduction remaining in some rivers, downstream of the lowest dam (Finnäs et al., 2020; Larsson et al., 2013; Leskelä et al., 1991). In these areas, eggs are subject to the hydropower stations' artificial flow regimes (see Ashraf et al., 2018; Bejarano et al., 2017), which have probably contributed to the low success rate of natural reproduction (Finnäs et al., 2020). Although dam removal would probably be the most efficient way to restore the reproduction success, such projects are often considered economically unfeasible under the increasing demands for non‐carbon‐based electricity, and therefore other mitigation measures are needed.

In the current study, our aim was to address this topic by assessing how water flow velocity and substrate type affect the downstream drift of whitefish eggs. The investigation was done using actual, fertilized whitefish eggs in artificial streams (flumes) under laboratory conditions. The aim was to provide knowledge on the effects of peak flow bouts from hydroelectric dams to assist decision making regarding regulated rivers, especially those that have surviving populations of anadromous *Coregonus* and other similar species.

## 2 METHODS

We carried out the study during November 28–December 4, 2023, at the Kainuu Fisheries Research Station, Paltamo (64°24′ N, 27°30′ E). We obtained fertilized *C*. *lavaretus* eggs for the experiment from the Kemijoki population (65°47′ N, 24°32′ E).

### 2.1 Experimental design

To assess the effects of the water flow velocity and substrate on the downstream drift of *Coregonus* eggs, we set up three identical drift flumes for the purpose. The overall length of the fiberglass flumes was 6 m, of which a 3‐m section was covered with substrate for the purpose of the experiment. We adjusted the width of the flumes to 25 cm with clay bricks on both sides, covered with 3‐mm plastic sheets for smooth water flow. Water level was set to 15–17 cm. The experiment had the following four substrate treatments: (1) cobble ~130 mm (hand‐measured D_50_ as per Garefalakis et al., [2023]; longest axis Q_1_ = 118 mm and longest axis Q_3_ = 147 mm), (2) coarse gravel ~50 mm (Q_1_ = 45 mm and Q_3_ = 69 mm), (3) gravel ~35 mm (Q_1_ = 33 mm and Q_3_ = 42 mm), and (4) sand ~1–2 mm. In each treatment, the volume of the material was similar, which resulted in a substrate thickness (~6 cm) that was more even with finer substrates and more variable in the case of the coarser ones. Besides the sand treatment, we used some sand (~12 L per replicate) also in all other treatments so that no bare flume bottom remained visible, and sand was added to the crevices that we subjectively considered the largest. We adjusted water velocity and water level by both manipulating (with valves) the inflow of river water (~4°C) into one end of the flume and obstructing water flow at its outflow end. We used a Schiltknecht MiniAir20 multiprobe anemometer, equipped with a water flow probe (range: 0.02–5 m/s and accuracy: ±2.0%), to measure the flow velocity a few centimeters below the surface multiple times during each phase of the experiment. In particular, the experiment consisted of five phases of increasing flow rate, with the nominal velocities of 0.05, 0.15, 0.30, 0.50, and 0.90 m/s. To assess the number of eggs that had drifted through the flume, we had, at the outflow end of the flume, a plastic net (mesh‐size 1000 μm, Sefar Nitex 06‐1000/57), which captured the eggs. We then counted the number of eggs in the net after each phase.

A replicate was initiated by adjusting the water flow to 0.05 m/s. We then placed *C*. *lavaretus* eggs within the first 50 cm of the substrate‐covered section of the flume (approximately in the middle of that section). In most replicates, we had 250 eggs. One replicate, however, had 350 eggs and a few others had 240–249 (due to technical challenges with the container from which the eggs were released). These egg number variations were accounted for in the statistical analyses (see “Statistical analyses,” below, for further details). After 10 min, we replaced the egg‐capture net with a new one and, within 1 min, increased the flow to 0.15 m/s. Similarly, 10 min after the new nominal flow rate was reached, we replaced the net and then increased the flow velocity to 0.30 m/s. The fourth phase had the flow velocity of 0.50 m/s. In the final, fifth, phase, in all treatments other than the sand substrate, we used the full flow capacity of the flume, which resulted in a variable flow that was on average ~0.90 m/s, depending on the point of measurement. In the sand substrate treatment, we were not able to use the full flow rate in the final phase, because that flow velocity was strong enough to flush large amounts of sand with it. Therefore, for the sand substrate type, we used 0.70 m/s in the final phase. We conducted *n* = 6 replicates for each of the four substrate‐type treatments. After a replicate was completed, we removed the substrate, flushed the bare flume, and set up a new replicate (with a different substrate type). We ran replicates in a haphazard order so that, at any given time, we had completed a similar number of replicates of each treatment.

To further estimate the potential of water flow to affect *Coregonus* eggs, we used vertical cylinders (ø 20 cm) to assess the time it took for a *C*. *lavaretus* egg to sink through 50 cm of a still‐water column. We then used these data to estimate the sinking rate as meters/minutes. We used the same eggs (eye‐spot stage) as in the main experiment. We repeated this assay 26 times.

### 2.2 Ethics statement

The experiment involved only fish eggs. Handling of the eggs complied with all relevant laws, guidelines, and policies. After the experiment, the eggs were terminated.

### 2.3 Statistical analyses

We ran all analyses using the R 4.2.2 software. We first bound (using the “cbind” command in R) the cumulative number of eggs that had drifted through the flume with the number of eggs that were still in the flume (at the end of the phase) to be used as a compound response variable. This way, our models (see below) assessed the proportions of eggs that had drifted through the flume while retaining the information about the absolute egg numbers. In the first model, we assessed differences between the substrate types over the entire dataset in a binomial generalized linear mixed model (GLMM), in which the substrate type and experimental phase (1–5) were denoted as explanatory variables and replicate ID as a random effect (to account for the repeated design with five phases in each replicate).

In the second set of models, we assessed at which point of the experiment treatment differences (if any) became apparent. For this purpose, we analysed the cumulative egg drift separately for each flow velocity phase by fitting phase‐specific generalized linear models (GLMs), each with a binomial distribution, the same response variable as above, and substrate type as the explanatory variable.

In the final set of models, we analysed egg drift within each phase. Here, we bound (using “cbind” in R) the number of eggs that drifted through the track during each phase with the number of eggs that had not yet drifted through by the end of that phase. This was then used as the response variable, and the substrate type was again the explanatory variable.

## 3 RESULTS

**FIGURE 1:**
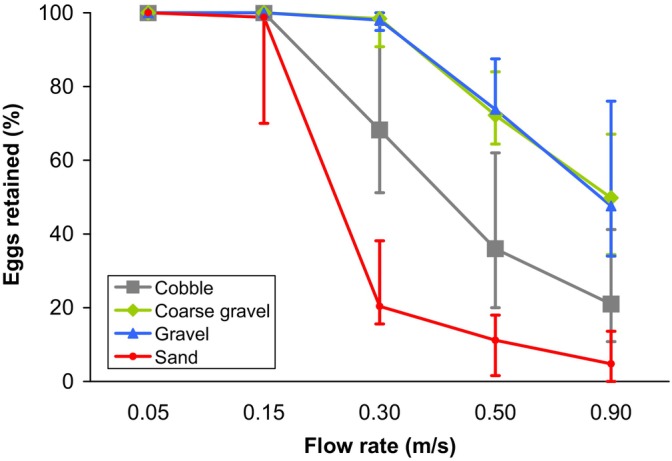
The proportion of eggs that had not flowed through the flume by the end of each flow rate phase. The error bars show the full range of observations, with *n* = 6 for each of the four substrate coarseness treatments. For practical reasons, the final flow rate was lower for sand (~0.70 m/s) than the other substrate types (0.90 m/s).

Due to the experimental design, the phase had a significant effect on egg drift in the first model, which included the entire dataset. In particular, the proportion of eggs that had drifted through increased from phase to phase (GLMM, phase effect: *β* = 1.762 ± 0.0224, *z* = 78.7, *p* < 0.001) (Figure 1). Regarding substrate types, egg drift through gravel and coarse gravel substrates was similar (Figure 1) and not significantly different between the two (GLMM, treatment comparison: *β* = 0.1602 ± 0.3063, *z* = 0.52, *p* = 0.60). In contrast, all other substrate types significantly differed from each other (GLMM, all other cases: *p* < 0.001), with sand allowing eggs to drift through most easily, followed by cobbles, while the two gravel types retained more eggs (Figure 1).

During the first two phases (0.05 and 0.15 m/s) of the experiment, very few eggs drifted through the flume, except for a moderate number of eggs drifting through in the sandy substrate treatment by the end of the second phase (Figure 1). By the end of the third phase (0.30 m/s), the pattern of egg drift followed that of the entire experiment (discussed above), with the sand substrate letting significantly more eggs through than other substrates, followed by cobbles and then the two gravel types (Figure 1; Table 1). Similarly in the remaining phases, the treatment differences were highly significant, except between gravel and coarse gravel (Table 1). In this regard, gravel had a modest tendency to retain more eggs than coarse gravel (Figure 1; Table 1).

**TABLE 1:** The results of the three generalized linear mixed models (GLMMs) that assessed treatment differences in cumulative egg drift in the last three phases of the experiment (0.30, 0.50, and 0.90 m/s).

Treatment comparison		0.30 m/s			0.50 m/s			0.90 m/s		
		*β* ± SE	*z*	*p*	*β* ± SE	*z*	*p*	*β* ± SE	*z*	*p*
Sand	Gravel	5.01 ± 0.19	27.0	**<0.001**	3.35 ± 0.10	32.6	**<0.001**	2.80 ± 0.12	23.3	**<0.001**
Sand	Coarse gravel	4.59 ± 0.16	28.7	**<0.001**	3.15 ± 0.10	30.9	**<0.001**	2.75 ± 0.12	22.7	**<0.001**
Sand	Cobble	2.10 ± 0.08	25.1	**<0.001**	1.68 ± 0.10	17.0	**<0.001**	1.59 ± 0.13	12.7	**<0.001**
Gravel	Coarse gravel	−0.42 ± 0.23	−1.84	0.066	−0.20 ± 0.08	−2.35	**0.019**	−0.05 ± 0.07	−0.76	0.45
Gravel	Cobble	−2.92 ± 0.19	−15.8	**<0.001**	−1.67 ± 0.08	−20.9	**<0.001**	−1.21 ± 0.08	−15.4	**<0.001**
Coarse Gravel	Cobble	−2.50 ± 0.16	−15.7	**<0.001**	−1.47 ± 0.08	−18.60	**<0.001**	−1.16 ± 0.08	−14.5	**<0.001**

*Note*: *p*‐Values < 0.05 are bolded.

**FIGURE 2:**
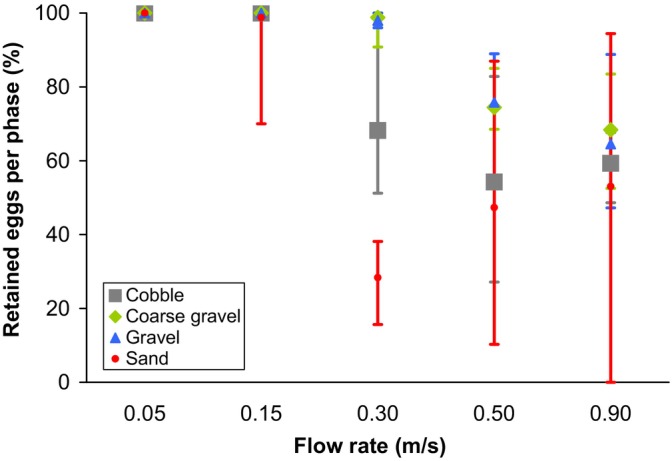
The proportion of eggs that did not flow through the flume during each phase (i.e., flow rate). The error bars show the full range of observations, with *n* = 6 for each of the four substrate coarseness treatments. For practical reasons, the final flow rate was lower for sand (~0.70 m/s) than the other substrate types (0.90 m/s).

When each phase was investigated separately, the general pattern remained similar but less pronounced (Figure 2; Table 2): in all phases from the third one (0.30 m/s) onward, sandy substrate let clearly the highest proportion of eggs through, followed by cobbles, whereas gravel and coarse gravel substrates retained higher proportions of eggs (Figure 2; Table 2). Differences in the numbers of eggs drifting through in the last phase (0.90 m/s) were less pronounced (Figure 2) and not significant between sand and cobbles (Table 2).

**TABLE 2:** The results of the generalized linear mixed models (GLMMs) that assessed treatment differences in egg drift within the last three phases of the experiment.

Treatment comparison		0.30 m/s			0.50 m/s			0.90 m/s		
		*β* ± SE	*z*	*p*	*β* ± SE	*z*	*p*	*β* ± SE	*z*	*p*
Sand	Gravel	4.97 ± 0.19	25.6	**<0.001**	1.53 ± 0.12	12.5	**<0.001**	0.44 ± 0.17	2.57	**0.010**
Sand	Coarse gravel	4.62 ± 0.16	27.4	**<0.001**	1.37 ± 0.12	11.1	**<0.001**	0.50 ± 0.17	2.91	**0.004**
Sand	Cobble	1.95 ± 0.08	23.2	**<0.001**	0.39 ± 0.12	3.20	**0.001**	0.20 ± 0.18	1.13	0.26
Gravel	Coarse gravel	−0.45 ± 0.24	−1.89	0.059	−0.17 ± 0.09	−1.90	0.057	0.06 ± 0.09	0.72	0.47
Gravel	Cobble	−3.02 ± 0.19	−15.6	**<0.001**	−1.14 ± 0.09	−13.1	**<0.001**	−0.23 ± 0.10	−2.25	**0.025**
Coarse Gravel	Cobble	−2.57 ± 0.16	−15.7	**<0.001**	−0.29 ± 0.11	−2.79	**0.005**	−0.29 ± 0.11	−2.79	**0.005**

*Note*: *p*‐Values < 0.05 are bolded.

In still water, the sinking rate of eye‐spot stage *C*. *lavaretus* eggs was 1.86 m/min (SE = 0.02, *n* = 26), which equals to 31 mm/s (SE = 0.3, *n* = 26).

## 4 DISCUSSION

We found that the coarseness of the substrate had a major effect on the drift of fertilized *Coregonus* eggs that were in the eye‐spot stage. In particular, gravel and coarse gravel retained eggs better than a coarser cobble substrate, whereas the sand substrate retained eggs poorer than the other substrates. This was evident by a notable proportion of eggs drifting already at 0.30 m/s when the substrate consisted of sand or cobble, whereas in the two different gravel substrate treatments, egg drift became pronounced only at 0.50 m/s. These differences between the substrates remained considerable also at the very fast flow of 0.90 m/s.

These results demonstrate that downstream drift of *Coregonus* eggs depends on both the flow velocity and riverbed substrate. Such findings imply that a river's characteristics, especially downstream of hydroelectric dams, affect the sensitivity of *C*. *lavaretus* eggs (and those of other similar species) to river flow fluctuations generated by dam operations (see also Schmutz et al., 2015). Whitefish eggs that drift away from the spawning site risk exposure to predation (by both fish and invertebrates), fungal attacks, burial by sediment, and other adverse physical, chemical, and biological conditions (Casas‐Mulet et al., 2015; Etheridge et al., 2011; Reshetnikov & Bogdanov, 2011; Ventling‐Schwank & Livingstone, 1994). Indeed, being swept away into unsuitable areas can be a major driver of egg mortality in *Coregonus* species (Murray et al., 2013; Reshetnikov & Bogdanov, 2011; Ventling‐Schwank & Livingstone, 1994).

Such egg drift can be a particularly serious issue to anadromous (i.e., river‐spawning) species. In most rivers running to the northern Baltic Sea, hydroelectric dams have greatly reduced or completely terminated natural reproduction of these populations by blocking the spawning migration upriver. Therefore, the anadromous *C*. *lavaretus* ecotype is maintained by extensive stocking programmes (e.g., Jokikokko & Huhmarniemi, 2014; Veneranta et al., 2024) and considered endangered (Urho et al., 2019). Accordingly, one of the aims of the study was to assess whether the success of natural reproduction could be improved by moderating the peak flows from the hydroelectric dams. The results suggest that high peak flow velocities can be an important factor contributing to the low success of natural reproduction, and that moderation of flow levels to <0.50 m/s during critical reproductive periods, in areas that are suitable for egg development, has potential to improve the situation.

We also found that *Coregonus* eggs sink at a moderate rate, which is faster than that of many other fish species (Robertson, 1981). *Coregonus* eggs are also thought to have characteristics that temporarily increase their adhesiveness (Murray et al., 2013), with the duration of adhesiveness seeming to be limited to a few hours (Veneranta, personal observations). Nevertheless, these two traits could be adaptive in the context of using sites with relatively high kinetic energy at the time of spawning, such as rivers and lake areas exposed to wave action. Our experiment involved eggs that had reached the eye‐spot stage and were first let to settle on the substrate. Therefore, newly laid eggs might be less likely to drift than the ones used in the current study. Even so, the results of the current study show that, when typical natural flow rates are exceeded, egg drift depends on the substrate, at least by the eye‐spot stage. The scale of our current experiment was too limited to provide exact estimations for the speed of the egg drift. Nevertheless, the results indicate that, within minutes, eggs may drift multiple meters.

It is relevant to note that our highest flow velocity was different for the sand substrate (~0.70 m/s) compared to the other substrate types (~0.90 m/s). The reason for treating sand substrate differently at the highest velocity phase was as much ecological as it was practical: we find it unlikely that sand substrates would persist at sites where the flow velocity approaches 1 m/s, and we were also unable to replicate such conditions in the laboratory without considerable loss of sand. Therefore, when reconditioning riverbeds below hydropower plants for fish reproduction, the effects of the plants' peak flow events on the substrate composition, in addition to fish eggs, should be considered. In the current study, the variability in particle size in all treatments was modest (except for the fact that some sand was used in all treatments). We therefore encourage future research that unravels the impact of variation in particle size on both substrate stability (Kirchner et al., 1990) and egg drift. Optimally, such investigations would be conducted in the field, where both mean particle size and its variation can have wider ranges than what was feasible to test here. Moreover, water current eddies in both natural and constructed rivers may differ from those occurring in the constrained scale of a flume setup. For instance, the impact of water depth on egg drift could not be simulated in the flumes, and therefore, we lack the information on how different flow characteristics on the riverbed versus higher in the water column might impact egg drift's speed and distance. *Coregonus* spawning below dams seems to take place at depths of a few meters (Veneranta, personal observations), but more detailed such knowledge is currently very limited. Therefore, we underscore the need to investigate the precise spawning sites in both natural and constructed rivers, as well as flow rate variations at different depths. In that regard, the current laboratory‐based results provide an important first step to understand the links between substrate coarseness and peak flow management needs in relation to egg drift.

To conclude, our laboratory experiment indicates that *Coregonus* eggs are prone to drift when water flow velocities are higher than 0.30 m/s, and the extent of this drift depends on both the velocity and the coarseness of the (riverbed) substrate. Therefore, mitigation of peak flows generated by hydropower plants requires an approach that considers the combined effects of the water flow and river “morphology”. Besides these, future assessments of fish reproduction in regulated rivers would benefit from including additional environmental factors, such as the changing climate and flood patterns (see Arheimer & Lindström, 2015; Blöschl et al., 2017; Parasiewicz et al., 2019). Regarding egg drift in particular, we also encourage field experiments that map the remaining natural reproduction and investigate the prerequisites for improving it. Here, the role of the substrate should be considered, not only from the perspective of how it affects the spawning adults, eggs, and larvae (Heikkilä et al., 2006; Lehtonen et al., 2024; Thome et al., 2020; Weidel et al., 2023) directly but also in the framework of flow velocity impacts on eggs, as highlighted by the current study.

## AUTHOR CONTRIBUTIONS

Lari Veneranta conceived the study and methodology, with Topi K. Lehtonen contributing to methodological details. Topi K. Lehtonen and Lari Veneranta collected the data. Topi K. Lehtonen analysed the data, designed the visualisations and wrote the first draft of the manuscript. Both authors provided edits to the manuscript and accepted its final version.

## FUNDING INFORMATION

A coalition of hydropower companies ('Sateenvarjo III' project).

## CONFLICT OF INTEREST STATEMENT

Topi K. Lehtonen and Lari Veneranta declare that they have no conflicts of interest.
